# Supplementary material for: The causal relationship between the human gut microbiota and pyogenic arthritis: a Mendelian randomization study
Source: Front Cell Infect Microbiol. 2024 Nov 26;14:1452480. doi: 10.3389/fcimb.2024.1452480 (PMC11629706; doi:10.3389/fcimb.2024.1452480)
Supplement: Supplementary file 1 [file DataSheet1.zip › mendelian test/ebi-a-GCST90027640.csv_leave_one_out.pdf]

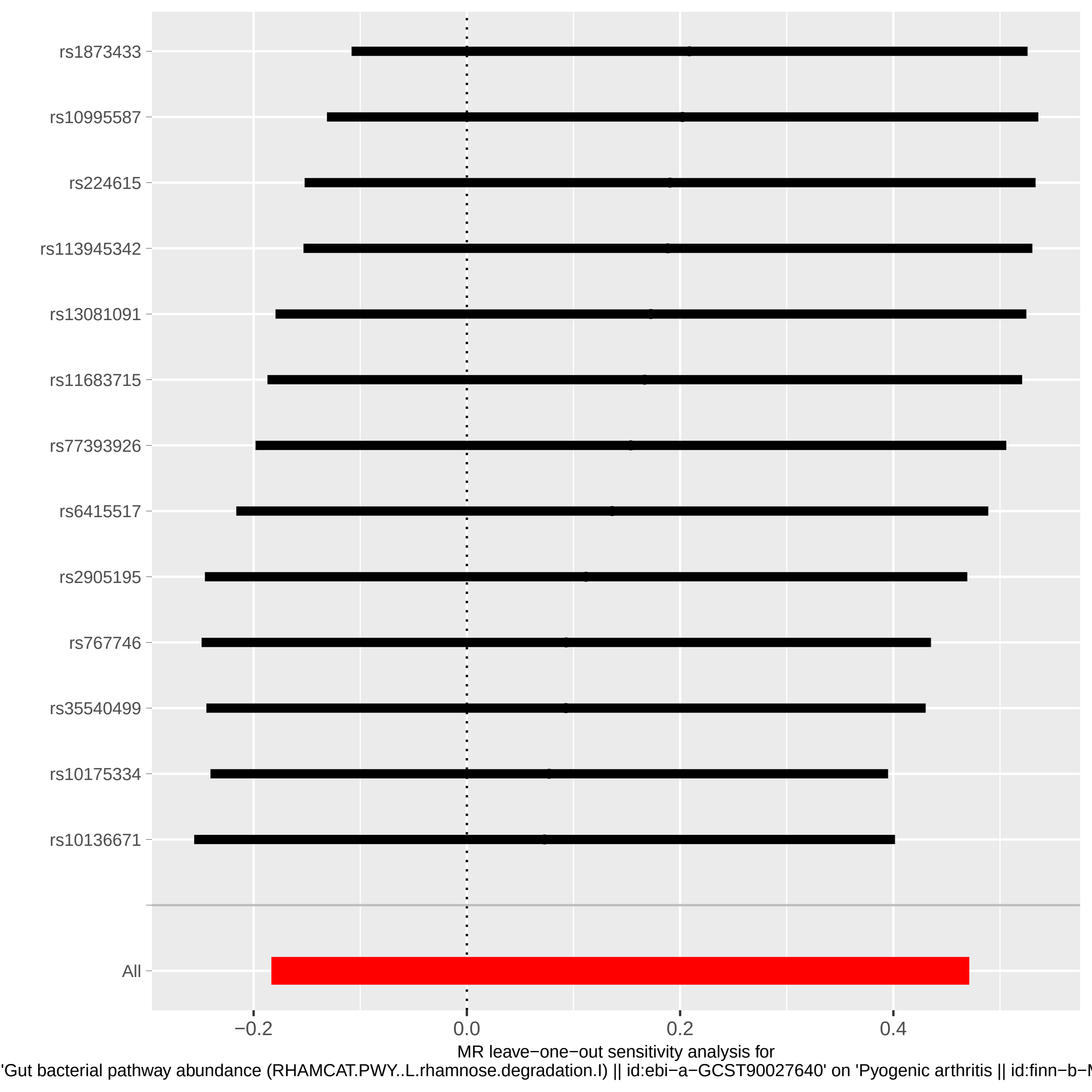
rs1873433
rs10995587
rs224615
rs113945342
rs13081091
rs11683715
rs77393926
rs6415517
rs2905195
rs767746
rs35540499
rs10175334
rs10136671
All
−0.2
0.0
0.2
0.4
MR leave−one−out sensitivity analysis for
'Gut bacterial pathway abundance (RHAMCAT.PWY..L.rhamnose.degradation.I) || id:ebi−a−GCST90027640' on 'Pyogenic arthritis || id:finn−b−M
